# Impaired Autophagic Flux in Adipose Tissue Aggravates Pancreatic Injury in Obesity‐Related Severe Acute Pancreatitis

**DOI:** 10.1002/iid3.70395

**Published:** 2026-03-09

**Authors:** Li‐Ping Sheng, Guo‐Chen Shang, Chao‐Qun Han, Xin Ling, Xian‐Wen Guo, Rong Lin, Zhen Ding

**Affiliations:** ^1^ Department of Gastroenterology, the First Affiliated Hospital of Xiamen University, School of Medicine Xiamen University Xiamen China; ^2^ Department of Gastroenterology, Union Hospital, Tongji Medical College Huazhong University of Science and Technology Wuhan China; ^3^ Department of Gastroenterology, the First Affiliated Hospital, School of Medicine Shihezi University Shihezi China; ^4^ Department of Gastroenterology, the First Affiliated Hospital Sun Yat‐sen University Guangzhou China

**Keywords:** adipose tissue, adipose tissue macrophages, impaired autophagic flux, lipid metabolism, severe acute pancreatitis

## Abstract

**Background:**

Adipose tissue (AT) playing a crucial role in obesity‐related pancreatitis. This study investigated the impact of autophagy in AT on pancreatic injury during obesity‐related severe acute pancreatitis (SAP).

**Methods:**

Non‐obese and obese mice were induced using a normal diet (ND) or high‐fat diet (HFD) before establishing SAP. Pancreatic injury and autophagy in AT were evaluated. Adipose tissue macrophages (ATMs) were analyzed. Autophagy in RAW 264.7 cells co‐cultured with palmitic acid (PA) and lipopolysaccharide (LPS), simulating the conditions of ATMs during SAP, was further investigated. The effect of PA on autophagy within inflammatory macrophages in vitro was explored, utilizing Sulfo‐N‐succinimidyloleate (SSO) to inhibit fatty acid transport. Additionally, the impact of autophagy in AT on inflammation in SAP mice was investigated using SSO, complemented by metabolomics analysis to uncover underlying molecular mechanisms.

**Results:**

Pancreatic histological scores were significantly higher in obese SAP mice than in non‑obese SAP mice (*n* = 6; 9.50 ± 1.05 vs. 7.33 ± 1.03; *p* = 0.0005). Impaired autophagic flux was observed in the AT of obese SAP mice compared with obese control mice, as indicated by increased LC3‑II (*p* = 0.0383) and p62 levels (*p* = 0.0171). PA induced impaired autophagic flux in inflammatory macrophages. Inhibition of fatty acid transport partially restored impaired autophagic flux in an ATM cell model of SAP. Furthermore, this inhibition also alleviated the impairment of autophagic flux in AT and attenuated pancreatic injury in obese SAP mice. Metabolomics analysis of AT revealed elevated levels of more fatty acids and several signaling pathways correlated with autophagy in obese SAP mice.

**Conclusion:**

Impaired autophagic flux in AT aggravates pancreatic injury in obese SAP mice, with impaired autophagic flux in ATMs likely playing a crucial role. Metabolomics analysis revealed abnormal lipid metabolism in AT of obese SAP mice, and several signaling pathways may contribute to impaired autophagic flux in this tissue.

## Summary

- Obesity exacerbates the severity of acute pancreatitis (AP), serving as significant contributing factor. Our study demonstrated that impaired autophagic flux in adipose tissue (AT) aggravated pancreatic injury in obese severe acute pancreatitis (SAP) mice.
- In obesity, adipose tissue macrophages (ATMs) are the predominant leukocytes in AT. Impaired autophagic flux in ATMs likely plays a crucial role in aggravating pancreatic inflammatory injury during SAP in obese mice.
- To explore the molecular mechanisms underlying impaired autophagic flux in AT of obese SAP, metabolomics analysis of AT in mice were performed. The results revealed that lipid metabolism in AT of obese SAP mice is abnormal, and several signaling pathways (HIF‐1, FoxO, and AMPK) likely contribute to impaired autophagic flux in this tissue. Targeting autophagy‐related pathways may offer a therapeutic strategy to attenuate inflammation in obesity‐related SAP.

## 1 Introduction

Acute pancreatitis (AP) is an inflammatory disorder of the pancreatic tissue that can progress to systemic inflammatory response syndrome and multi‐organ dysfunction [1]. The severity and mortality rates of AP are notably exacerbated by obesity [2, 3]. Current research highlights that increased severity in AP and a higher occurrence of local complications are associated with coexisting hypertriglyceridemia and abdominal obesity [4]. This condition presents significant clinical challenges. However, the underlying mechanisms through which obesity exacerbates AP injury remains unclear.

Obesity results in the accumulation of adipose tissue (AT), which is characterized by low‐grade inflammation [5, 6]. This inflammation is further exacerbated by AP, which generates numerous mediators that may induce a systemic inflammatory response. Specifically, regions of fat necrosis during AP are key contributors to the production of inflammatory mediators [7]. Adipose tissue macrophages (ATMs) are the dominant leukocytes in AT under obese conditions [8]. ATMs have been shown to amplify inflammation during severe acute pancreatitis (SAP) in obese mice [9, 10]. To explore the mechanisms by which ATMs exacerbate inflammation in SAP under obesity, we focused on impaired autophagy, which has been implicated in various diseases [11].

Our study demonstrated that impaired autophagic flux in AT aggravated pancreatic inflammatory injury during SAP in obese mice, with ATMs likely playing a significant role. To explore the potential molecular mechanisms, metabolomics analysis of AT in mice were performed. The significance of our study lies in its potential to enhance the mechanistic understanding of how obesity impacts the severity of AP, thereby providing valuable insights for researchers examining the association between inflammation and metabolic diseases.

## 2 Materials and Methods

### 2.1 Ethics Declaration

All experimental protocols were approved by the Institutional Animal Care and Use Committee, Huazhong University of Science and Technology (IACUC No. 3579, [2021]).

### 2.2 Animal Experiment Design and Procedure

Male C57BL/6JNifdc mice, aged 4 weeks, were obtained from Vital River Laboratories (Beijing, China). The mice were fed either a high‐fat diet (HFD) or a normal diet (ND) for more than 16 weeks. The HFD (60% kcal from fat) was purchased from Ruidi Biotechnology Co. Ltd (Shenzhen, China), while the ND (approximately 12% kcal from fat) was provided by Experimental Animal Center of Tongji Medical College, Huazhong University of Science and Technology. All mice were kept in a specific‐pathogen‐free and temperature‐controlled environment with a cyclic light schedule.

Before the induction of SAP, the mice underwent an overnight fast. SAP was induced through 7 hourly intraperitoneal (IP) administrations of cerulein (50 μg/kg), followed by a single IP injection of lipopolysaccharide (LPS) (10 mg/kg). Sulfo‐N‐succinimidyloleate (SSO) was used as a CD36 inhibitor to block fatty acid uptake [12, 13]. Mice were administered SSO (50 μg/g) (Toronto Research Chemicals, Toronto, Canada) by gavage 0.5–1.5 h prior to cerulein administration. In the control groups, animals received normal saline. Mice were randomized using a computer‐generated random number sequence. A total of 72 mice were used in this study, with 6 mice per group. The experiment was divided into two parts. Part one included 24 mice and consisted of four groups: ND + normal control (NC), ND + SAP, HFD + NC, and HFD + SAP. Part two involved 48 mice subjected to SSO treatment and comprised eight groups: ND + NC, ND + NC + SSO, ND + SAP, ND + SAP + SSO, HFD + NC, HFD + NC + SSO, HFD + SAP, and HFD + SAP + SSO.

Mice were euthanized more than 22 h after the LPS injection, and pancreatic tissue and AT were immediately collected for further analysis. Epididymal white AT was specifically used in this study [14].

### 2.3 Histological Assessment

The pancreas was preserved in 4% paraformaldehyde, followed by paraffin embedding and sectioning into 3–5 μm slices. Hematoxylin and eosin (H&E) staining was performed on the tissue sections. An optical microscope (BX53F or IX73, Olympus, Tokyo, Japan) was used to visualize pancreatic tissue damage. Histopathological scoring for edema, inflammatory cell infiltration, vacuolization, and necrosis was conducted by an experienced researcher following referenced protocols [15, 16].

### 2.4 Cell Treatments

RAW 264.7 cells were co‐cultured with palmitic acid (PA) and LPS to simulate the conditions of ATMs during SAP. The cells were exposed in vitro to 0.1 μg/mL LPS and/or 100 μM PA (MedChemExpress, Monmouth Junction, USA) for 12–13 h. The PA stock solution was prepared in a solvent containing 20% bovine serum albumin (BSA) (fatty acid‐depleted, IgG‐free, Beyotime Biotechnology, Shanghai, China) and 0.05 M NaOH. A vehicle control consisted of 20% fatty acid‐free/IgG‐free BSA and 0.05 M NaOH. Prior to PA and LPS exposure, the cells were pre‐incubated with 500 μM SSO for 5–6 h.

### 2.5 Western Blot Analysis

Proteins were extracted from AT or RAW 264.7 cells. Following quantification and denaturation, equal amounts of protein (20–30 μg) were resolved by electrophoresis using 10% or 12.5% sodium dodecyl sulfate polyacrylamide gel electrophoresis gels. After electrophoretic transfer to polyvinylidene fluoride membranes, blocking was performed with either a conventional blocking buffer (1 h, room temperature) or a rapid blocking buffer (15–30 min, room temperature). Membranes were incubated with primary antibody overnight at 4°C: anti‐p62 (1:10,000; ab109012, Abcam, Cambridge, UK), anti‐LC3B (1:2,000; ab192890, Abcam, Cambridge, UK), and anti‐GAPDH (1:2,000; AC002, ABclonal, Wuhan, China). After primary antibody incubation, membranes were incubated with a 1:2000 to 1:3000 dilution of secondary antibody (AntGene, Wuhan, China) at room temperature for 1 h. Protein bands were visualized using a chemiluminescence imaging system (ChemiScope 6200, Clinx, Shanghai, China). Protein measurements were performed using ImageJ software.

### 2.6 Metabolomics Analysis of AT in Mice

Metabolite profiling in AT was conducted using liquid chromatography‐tandem mass spectrometry (LC‐MS/MS) on an SCIEX LC‐MS QTRAP 6500+ analytical instrument. Three samples for three mice were included in each group. HMQuant software (Beijing Genomics Institution, BGI) was used with default settings to automatically recognize and integrate each multi‐reaction monitoring transition, followed by manual verification. Different analysis on LC‐MS/MS data was performed using the R package metaX. A volcano plot was used to illustrate the differences in metabolite quantitative values between the two groups and the statistical significance of these differences. The metabolomics analysis was performed with only three biological replicates per group. To avoid overlooking potentially meaningful but modest effects in this exploratory study, differential metabolites were identified based on a fold change (FC) of ≥ 1.2 or ≤ 0.83 and an unadjusted two‐sided t‐test *p*‐value < 0.05. Metabolites satisfying the stricter criterion of False Discovery Rate‐adjusted *p* < 0.05 and FC of ≥ 1.2 or ≤ 0.83 are listed in the Supporting Materials. Enrichment analysis of metabolic pathways for differential metabolites was performed against the Kyoto Encyclopedia of Genes and Genomes (KEGG) database using over‐representation analysis. Significantly enriched pathways were visualized as bubble plots.

### 2.7 Statistical Analysis

All data, except for the LC‐MS/MS results, were analyzed as described below. Statistical analyses were performed using GraphPad Prism 8.2.1. Continuous data are presented as mean ± standard deviation (SD). Normality was assessed with the Shapiro–Wilk test and homogeneity of variances with the Brown–Forsythe test. If assumptions of normality and equal variances were met, group differences were evaluated by one‑way analysis of variance (ANOVA) with Sidak's multiple‑comparisons test. If variances were unequal, Welch's ANOVA was applied with Dunnett's T3 post hoc comparisons. For variables that could not be made approximately normal, nonparametric analyses (Kruskal–Wallis test with Dunn's post hoc multiple comparisons) were used. Two‐sided *p* < 0.05 was considered statistically significant. Experiments were conducted with at least three replicates.

## 3 Results

### 3.1 Obesity Exacerbated Pancreatic Injury in SAP Mice

**Figure 1:**
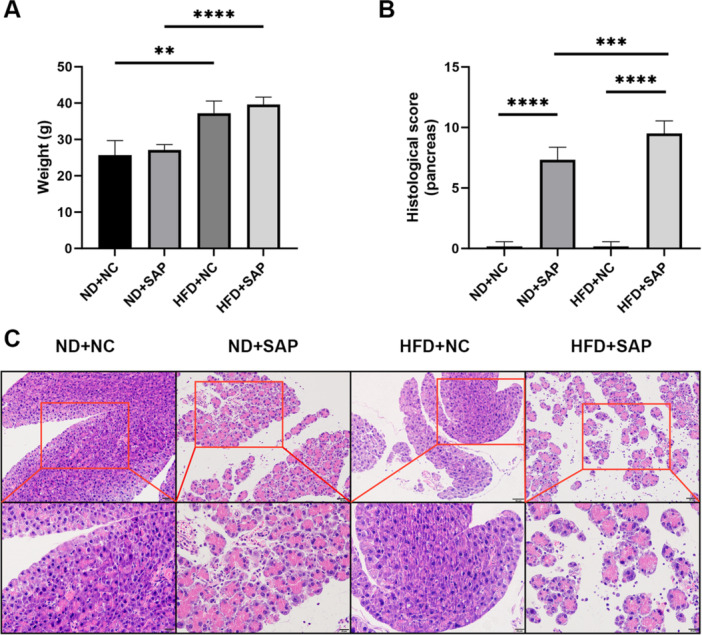
Obesity exacerbated pancreatic injury in SAP mice. (A) Obesity was induced in mice through HFD (**p = 0.0011, ****p < 0.0001). (B) Obesity increases histological score of pancreatic injury, scoring for edema, inflammation, vacuolization, and necrosis (*R* ^2^ = 0.9713, ***p = 0.0005, ****p < 0.0001). (C) Representative photomicrographs depict H&E‐stained pancreatic slices, with a scale bar of 50 µm for first row and 20 µm for second row.

SAP mice on a HFD gained significantly more weight than SAP mice on a ND (*p* < 0.0001, Figure 1A). Following the induction of SAP, pancreatic injury occurred in both ND‐fed and HFD‐fed mice. The injury scores for the HFD + SAP group were significantly higher than those for the ND + SAP group (*p* = 0.0005, Figure 1B,C). These findings suggest that obesity exacerbates pancreatic injury in the context of SAP.

### 3.2 Impaired Autophagic Flux in AT of Obese SAP Mice

**Figure 2:**
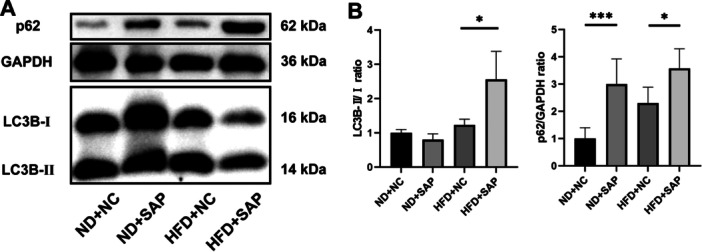
Impaired autophagic flux in AT of obese SAP mice. Western blot (A) and quantification (B) of LC3‐II (*p = 0.0383) and p62 (*R* ^2^ = 0.7022, *p = 0.0171, ***p = 0.0003) protein levels.

The HFD + SAP group exhibited significantly higher levels of LC3‐Ⅱ and p62 (autophagic flux markers) compared to the HFD + NC group (*p* = 0.0383 and *p* = 0.0171, respectively; Figure 2). This suggests a blockage of autophagic flux in the AT of obese SAP mice. The impaired autophagic flux in AT may contribute to the exacerbated pancreatic injury observed in obese SAP mice. We further investigated autophagic flux in ATMs in vitro, as they are the predominant immune cells in AT during obesity.

### 3.3 PA Induced Impaired Autophagic Flux in Inflammatory Macrophages

**Figure 3:**
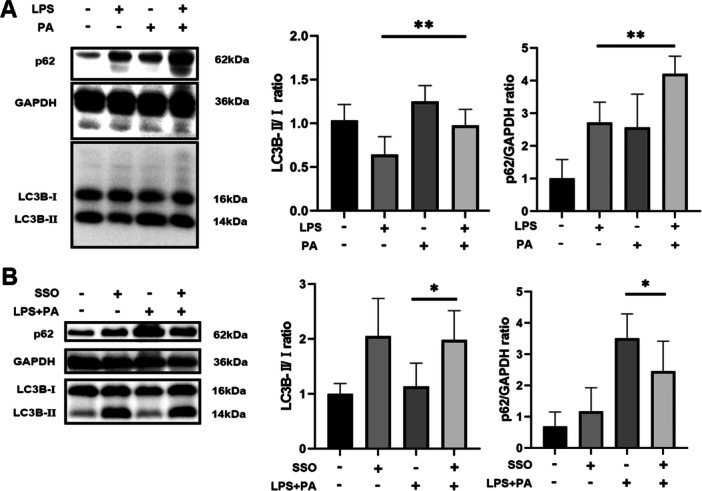
PA Induced impaired autophagic flux in inflammatory macrophages. (A) PA induced impaired autophagic flux in LPS‐stimulated RAW 264.7 cells (*R* ^2^ = 0.6179, **p = 0.0061 for LC3‐Ⅱ; *R* ^2^ = 0.7495, **p = 0018 for p62). (B) SSO, which inhibits fatty acid transport, alleviated the PA‐induced impairment of autophagic flux in inflammatory RAW 264.7 cells (*R* ^2^ = 0.5537, *p = 0.0323 for LC3‐Ⅱ; *R* ^2^ = 0.7181, *p = 0.0267 for p62). The protein expression levels of LC3‐Ⅱ and p62 were evaluated via Western blotting analysis.

ATMs are key immune cells in AT during obesity. To simulate ATMs conditions during SAP, RAW 264.7 cells were co‐cultured with PA and LPS. Then, autophagic flux in the cells was investigated. Inflammatory RAW 264.7 cells induced by LPS showed a significant upregulation of LC3‐Ⅱ and p62 levels upon treatment with PA, compared to untreated inflammatory RAW 264.7 cells (*p* = 0.0061 and *p* = 0018, respectively; Figure 3A). SSO, which blocks fatty acid transport, increased LC3‐Ⅱ levels and decreased p62 levels in RAW 264.7 cells stimulated with PA and LPS, compared to cells incubated only with PA and LPS (*p* = 0.0323, *p* = 0.0267, respectively; Figure 3B). These results suggest that PA induces impaired autophagic flux in inflammatory macrophages.

### 3.4 Inhibition of Fatty Acid Transport Ameliorated Impaired Autophagic Flux in AT and Inflammation in Obese SAP Mice

**Figure 4:**
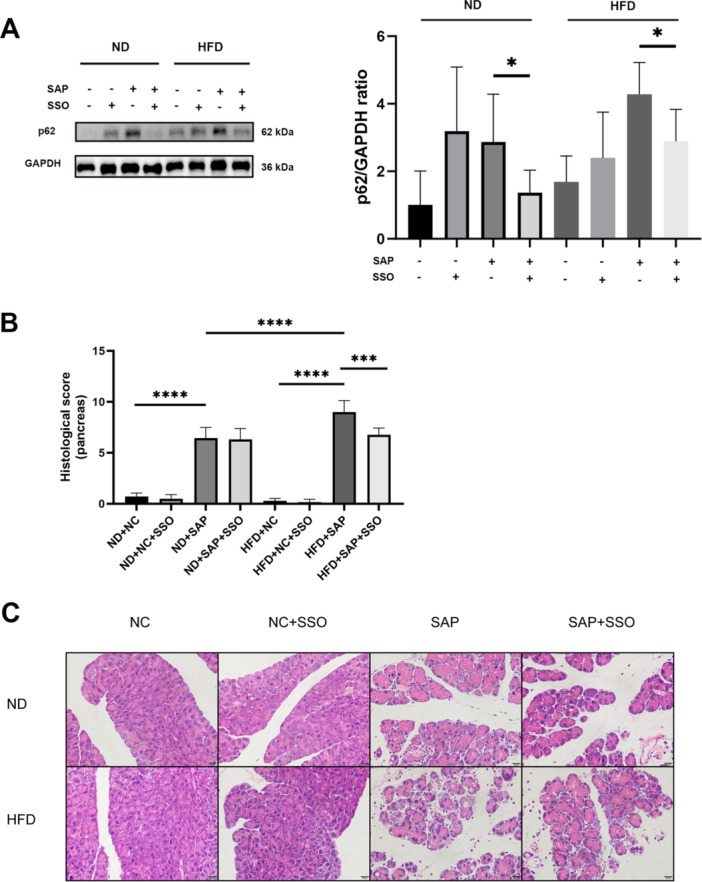
Blocking fatty acid transport ameliorated impaired autophagic flux in AT and inflammation in obese SAP mice. (A) SSO, which inhibits fatty acid transport, decreased protein levels of p62 in AT of SAP mice (*p = 0.0292 for ND mice; *p = 0.0123 for HFD mice). (B) SSO reduced the histological score of pancreatic injury in obese SAP mice, scoring for edema, inflammatory cell infiltration, vacuolization, and necrosis (*R* ^2^ = 0.9634, ***p = 0.0001, ****p < 0.0001). (C) Photomicrographs of H&E‐stained pancreatic slices, with a scale bar of 20 µm.

SSO significantly reduced the p62 levels, improving impaired autophagic flux, in AT of obese SAP mice compared to untreated obese SAP mice (*p* = 0.0123, Figure 4A). Furthermore, SSO treatment significantly decreased the histological score of pancreatic injury in obese SAP mice compared to untreated counterparts (*p* = 0.0002, Figure 4B). Characteristic histological changes in the pancreas of all groups are shown in Figure 4C.

### 3.5 Metabolic Abnormalities in AT of SAP Mice

**Figure 5:**
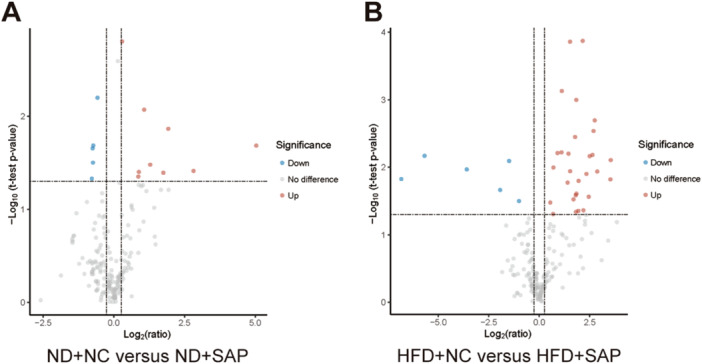
Metabolic abnormalities in AT of SAP mice. (A) Volcano plot of metabolites between ND+NC and ND+SAP. (B) Volcano plot of metabolites between HFD+NC and HFD+SAP. The horizontal axis indicates log_2_(ratio), while the vertical axis represents −log_10_(*p*‐value). Metabolites with a ratio of ≥ 1.2 or ≤ 0.83 and a *p*‐value < 0.05 are marked in red or blue, indicating up‐regulation and down‐regulation, respectively, while other metabolites are marked in gray.

Metabolomics analysis revealed significant metabolic abnormalities in AT of SAP mice. Specifically, 29 metabolites were upregulated and 6 downregulated in the HFD + SAP group relative to the HFD + NC group. In contrast, the ND + SAP group exhibited only nine upregulated and five downregulated metabolites compared to the ND + NC group. The differential metabolites volcano plots are shown in Figure 5. There are more differential metabolites between the HFD + NC and HFD + SAP groups than between the ND + NC and ND + SAP groups, highlighting significant metabolic disturbances in obese SAP. The number of elevated fatty acids was greater between HFD + NC and HFD + SAP groups than between ND + NC and ND + SAP groups, indicating lipid metabolism disruptions associated with obese SAP.

### 3.6 Metabolomics Analysis Revealed Autophagy‐Related Pathways in AT of Obese SAP Mice

**Figure 6:**
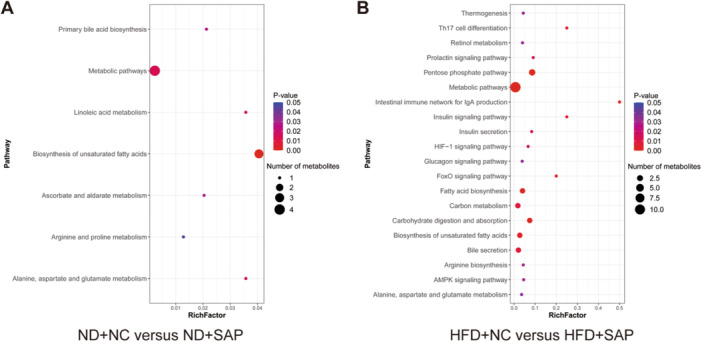
Metabolomics analysis revealed autophagy‐related pathways in AT of obese SAP mice. (A) Bubble plot showing pathways significantly enriched with differential metabolites between ND+NC and ND+SAP. (B) Bubble plot showing pathways significantly enriched with differential metabolites between HFD+NC and HFD+SAP. A *p*‐value of < 0.05 indicates significant enrichment. The enrichment factor on the horizontal axis indicates the proportion of differential metabolites linked to the pathway relative to all identified metabolites in that pathway. A greater value indicates a larger proportion of differential metabolites. The count of differential metabolites associated with the pathway is reflected in the bubble size.

Pathway analysis using the KEGG database identified many signaling pathways that were significantly enriched with differential metabolites. A broad range of these pathways are depicted in the bubble plot (Figure 6). Literature review revealed that several of signaling pathways between the HFD + NC and HFD + SAP groups were correlated with autophagy, including the hypoxia‐inducible factor 1 (HIF‐1), forkhead box O (Foxo), and AMP‐activated protein kinase (AMPK) signaling pathways [17, 18, 19]. Alteration in these pathways may play a crucial role in the impaired autophagic flux observed in AT of obese SAP mice.

## 4 Discussion

Pancreatitis significantly affects patients' health, and the timing of intervention is crucial for improving outcomes [20, 21]. Obese individuals are at a higher risk of developing AP and experiencing increased disease severity [22]. Our study demonstrated that obesity, induced by HFD, was closely associated with heightened pancreatic injury. Specifically, our study found that pancreatic damage was more severe in the obese SAP group compared to the non‐obese SAP group, consistent with previous research [9, 10].

In this study, we found impaired autophagic flux in AT of obese SAP mice. Pharmacological inhibition of fatty acid transport alleviated the impairment of autophagic flux in AT and attenuated inflammatory injury in obese mice with SAP. These results indicate that impaired autophagic flux in AT aggravates inflammatory injury in obesity‐related SAP mice.

In obesity, ATMs are the most prevalent type of leukocytes found in AT [8]. Moreover, macrophage autophagy regulates inflammasome activation and the release of inflammatory factors [23]. In obese mice, impaired macrophage autophagy promotes proinflammatory macrophage polarization, enhancing the immune response [24]. Therefore, impaired autophagic flux in ATMs is likely an important factor contributing to the aggravation of inflammatory injury in obese mice with SAP. Our study found that in obese SAP mice, impaired autophagic flux in AT exacerbated pancreatic inflammation, with disrupted autophagic flux in ATMs likely playing a significant role. AT inflammation, as a secondary process to AP, contributes to the production of mediators, with fat necrosis regions being key sources of these inflammatory mediators [7]. In obese individuals, the number of ATMs increases and participates in activated inflammatory pathways in AT [25]. M1‐polarized ATMs amplify inflammatory responses in obese SAP mice [9]. Additionally, inflammation in ATMs of obese SAP mice is enhanced through the NLRP3‐caspase1 inflammasome pathway [10].

To explore the probable molecular mechanisms of impaired autophagic flux in AT of obese SAP, metabolomics analysis of AT in mice was performed. We detected more differential metabolites between the obese SAP and obese control groups. Moreover, we observed lipid metabolism disruptions in AT in obese SAP mice. Based on KEGG database, the bubble plot for pathways with significantly enriched differential metabolites revealed various signaling pathways. Through literature reviewing, we identified several signaling pathways correlated with autophagy, including the HIF‐1, FoxO, and AMPK signaling pathways in obese SAP.

The results of metabolomics analysis revealed abnormal lipid metabolism in the AT of obese SAP mice, with an increased number of elevated differential fatty acids. HIF‐1α acts as a regulatory factor in autophagy and is a critical downstream target of fatty acids [17]. The HIF‐1α/AMPK signaling pathway is associated with impaired autophagy [26]. Moreover, TFEB and TFE3 play vital roles in regulating autophagy and lysosomal processes, with AMPK controlling their transcriptional activity [19]. As a key downstream target of AMPK, mTOR acts as a negative regulator of autophagy [27]. Furthermore, FoxO transcription factors play a multifaceted role in autophagy [18]. Specifically, FOXO1 promotes the nuclear import of FOXO3, which stimulates the upregulation of ATG3, ultimately inducing autophagy [28].

## 5 Conclusion

Our study revealed that impaired autophagic flux in AT exacerbated inflammatory injury in obese mice with SAP. ATMs are predominant leukocytes in AT during obesity, and their impaired autophagic flux likely plays an important role in aggravating inflammatory injury in obese mice with SAP. Metabolomics analysis indicates that lipid metabolism in AT of obese SAP mice is abnormal. It also suggests that several signaling pathways, including HIF‐1, FoxO, and AMPK, may contribute to impaired autophagic flux in AT of obese SAP mice. These findings highlight the potential of targeting autophagy‐related pathways as a therapeutic approach to alleviate inflammation in obesity‐associated SAP.

## Author Contributions

**Li‐Ping Sheng:** writing – original draft. **Guo‐Chen Shang:** writing – original draft. **Chao‐Qun Han:** writing – original draft. **Xin Ling:** methodology. **Xian‐Wen Guo:** methodology. **Rong Lin:** methodology. **Zhen Ding:** methodology.

## Conflicts of Interests

The authors declare no conflicts of interest.

## Supporting information

Supporting materials.

## Data Availability

We provide detailed information on differential metabolites identified in the Supporting Materials. Upon reasonable request, the corresponding author will provide the supporting data for the study.
